# Cateteres venosos totalmente implantáveis: histórico, técnica de implante e complicações

**DOI:** 10.1590/1677-5449.008216

**Published:** 2017

**Authors:** Antonio Eduardo Zerati, Nelson Wolosker, Nelson de Luccia, Pedro Puech-Leão

**Affiliations:** 1 Universidade de São Paulo – USP, Faculdade de Medicina, Hospital das Clínicas, São Paulo, SP, Brasil.

**Keywords:** artigo histórico, cateteres, dispositivos de acesso vascular, infecção, ultrassonografia de intervenção

## Abstract

O acesso ao sistema venoso, seja para coleta de amostras de sangue ou para infusão de soluções, é de vital importância para o diagnóstico e tratamento de pacientes com as mais variadas condições clínicas. Desde que Harvey, em 1616, descreveu o sistema circulatório a partir de estudos em animais e que Sir Christopher Wren, 4 décadas depois, realizou a primeira infusão endovenosa em seres vivos, a evolução na técnica de acesso e nos dispositivos para infusão tem sido constante. Merece destaque a criação dos cateteres de longa duração na década de 1970, em especial os totalmente implantáveis, que revolucionaram o tratamento do câncer, aumentando a segurança e o conforto dos pacientes oncológicos. Este artigo tem como objetivo a revisão de dados históricos relativos ao acesso vascular e a discussão da técnica de implante e das principais complicações associadas ao procedimento de colocação e ao uso dos cateteres totalmente implantáveis.

## HISTÓRICO

A fisiologia dos vasos sanguíneos passou a ser desvendada no século XVII por Harvey, que, em 1616, realizou pesquisas em animais e descreveu o sistema circulatório em *Excercitatio Anatomica de Moto Cordis et Sanguinus in Animalbus* 1. Esses conhecimentos permitiram que, passadas algumas décadas, fosse possível realizar intervenções nos vasos sanguíneos de seres vivos, como fez Folly em 1654, quando procedeu à primeira transfusão sanguínea entre dois animais através de um tubo de prata inserido na artéria do doador e uma cânula óssea inserida na veia do receptor^2^.

Em 1656, Sir Christopher Wren, conhecido como o arquiteto da St Paul’s Cathedral, realizou a primeira infusão no sistema venoso de seres vivos ao administrar ópio, cerveja e vinho no interior da veia de cães, utilizando para isso uma pena de ganso conectada a uma bexiga suína^3^.

Robert Boyle, em 1663, e Richard Lower, em 1667, relataram transfusão de sangue de animais em humanos^4^. A primeira transfusão sanguínea entre seres humanos se deu apenas em 1818 graças a Blundell^5^, que transfundiu para uma paciente em choque hemorrágico pós-parto o sangue extraído de outro indivíduo.

O’Shaughnessy^6^, em 1831, e Latta^7^, no ano seguinte, obtiveram sucesso no tratamento de doentes com cólera através da infusão de solução salina endovenosa, mesmo princípio descrito para o tratamento de indivíduos em choque^8^.

O primeiro cateter de polietileno introduzido por punção através do lúmen de uma agulha foi criado em 1945, passando a ser comercializado com o nome de Intracath® (BD Worldwide, Franklin Lakes, New Jersey)^9^.

O acesso ao sistema venoso por punção foi criado pelo cirurgião militar francês Robert Aubaniac, que descreveu a técnica em 1952^10^. A punção da veia subclávia por ele relatada permitia a infusão de maiores volumes de fluidos mais rapidamente para o tratamento de indivíduos em choque hipovolêmico nos campos de batalha. A técnica descrita por Aubaniac envolvia um acesso medial, dirigindo então a punção lateral e inferiormente em direção à fossa adjacente ao esterno. Dissecções *postmortem* mostraram que o sítio de entrada dos cateteres na veia subclávia ocorria próximo à junção com a veia jugular interna^10^.

Em 1952, Seldinger^11^ descreveu a inserção intravascular de cateteres avançando-os por navegação através de um fio-guia flexível introduzido por punção. Essa técnica é a base para o acesso nos procedimentos realizados por via endovascular atualmente.

A inserção de cateteres centrais por veias periféricas dos membros foi descrita por Wilson, em 1960, e tinha como finalidade monitorar a pressão venosa central de pacientes críticos^12^.

O acesso supraclavicular percutâneo à veia subclávia foi descrito em 1965 por Yoffa^13^. Nessa época, outras técnicas de cateterização percutânea das veias jugulares interna e externa já eram usadas^3^.

O avanço nas vias de acesso de longa duração teve início em 1973, quando Broviac criou um cateter de silicone que era exteriorizado pela parede anterior do tórax após tunelização subcutânea a partir do local de punção. Esse dispositivo era sintetizado em silicone e portava um anel de poliéster que, por provocar reação inflamatória, proporcionava melhor fixação do cateter, devido à aderência desse anel ao tecido subcutâneo^14^.

Em 1979, Hickman adaptou o dispositivo de Broviac, criando um novo modelo mais calibroso que permitia a realização de plasmaferese e o transplante de medula óssea (TMO)^15^.

Outro grande passo na evolução dos acessos vasculares deve-se à criação dos cateteres totalmente implantáveis. Essa modalidade surgiu no início dos anos 1970, quando Belin et al.^16^, em 1972, descreveram o implante de um cateter venoso central (CVC) com câmara subcutânea para infusão de nutrição parenteral. Em 1982, Niederhuber et al.^17^ mostraram os resultados de experimentos com 30 dispositivos totalmente implantáveis para tratamento de pacientes com câncer, sendo 20 com a extremidade em posição venosa central e os demais com implante arterial. Esses cateteres totalmente implantáveis são hoje largamente utilizados, principalmente no tratamento oncológico, constituindo o tópico deste estudo.

## ACESSOS VASCULARES EM PACIENTES ONCOLÓGICOS

A escolha do acesso vascular, para que seja capaz de proporcionar conforto e segurança ao paciente, deve considerar diversos fatores, tais como a definição de quais drogas serão ministradas, qual o tempo previsto de duração do tratamento, a frequência de uso do acesso, necessidade de transfusão de hemoderivados e condição da rede venosa periférica do indivíduo.

## TIPOS DE CATETERES

Os diferentes tipos de acesso venoso podem ser classificados em relação ao tempo de uso, frequência de uso e localização de sua extremidade (Tabela 1).

**Tabela 1:** Classificação dos tipos de cateter mais utilizados.

**Cateter**	**Duração**	**Inserção/posição da extremidade**	**Frequência de uso**
Jelco®	Até 4 dias	P/P	Contínuo
CVC de curta duração	Até 3 semanas	C/C	Contínuo
PICC	Até 12 meses	P/C	Contínuo/Intermitente
Semi-implantáveis	Meses a anos	C/C	Contínuo/Intermitente
Totalmente implantáveis	Anos	C, P/C	Intermitente

P: periférica; C: central; PICC: cateter central de inserção periférica; CVC: cateter venoso central.

Os cateteres periféricos de curta duração são fabricados em *teflon* ou silicone, têm cerca de 35 a 52 mm de comprimento e são inseridos por punção de veias periféricas, num procedimento de baixo risco. Têm custo reduzido e durabilidade curta, sendo os mais utilizados na prática clínica em pacientes internados.

Cateteres venosos centrais de curta duração são dispositivos de poliuretano com 20 a 30 cm de comprimento e calibre de até 8 Fr, passados por punção de uma veia central (jugular interna, subclávia, axilar ou femoral) e com a ponta posicionada próximo à junção átrio-cava. Há versões de lúmen único ou múltiplo, sempre para uso contínuo exclusivamente em pacientes sob regime de internação hospitalar. Seu uso domiciliar é desaconselhável, devido ao maior risco de infecção e de deslocamento do dispositivo que, por não ser tunelizado, fica fixo apenas por um ponto de fio inabsorvível que o fixa à pele junto ao orifício de entrada. O modelo mais calibroso (12 Fr), conhecido como Schilley, permite alto fluxo, necessário em sessões de hemodiálise ou aférese, com a ressalva de que tem curta duração.

Os cateteres centrais de inserção periférica (PICCs, do inglês *peripherally inserted central catheters*), são inseridos também por punção de veia superficial, geralmente do membro superior (antecubital, basílica, cefálica), ou com auxílio de ultrassonografia (US), também por punção da veia braquial. São cateteres não tunelizados, porém de longa duração, cuja ponta é mantida em posição central. Seu uso pode ser contínuo ou intermitente, nos pacientes em tratamento domiciliar ou internados. O procedimento de inserção representa baixo risco e pode ser feito à beira do leito, com o inconveniente de não haver controle de imagem durante a progressão do cateter. Por ser longo (50 a 65 cm de comprimento) e pouco calibroso (até 5 Fr), não é o cateter adequado para a infusão de grandes volumes em curto espaço de tempo. Tem a vantagem de ser facilmente removível, porém traz desvantagens em relação a questões estéticas e de conforto.

Cateteres tunelizados têm maior durabilidade, uma vez que o trajeto subcutâneo é fator protetor contra infecções^18^, além de proporcionar melhor fixação do dispositivo^19^. Os cateteres semi-implantáveis são introduzidos a partir de um orifício de entrada na pele (geralmente da parede anterior do tórax) e passados por um trajeto subcutâneo até o sítio de introdução numa veia central, ponto este a partir do qual adentra o espaço intravascular até que sua extremidade atinja a posição próxima à junção átrio-cava. Há dois tipos principais de cateteres semi-implantáveis: um modelo mais maleável e com ponta simétrica dos lúmens (geralmente dois), conhecido como Hickman, e outro de maior rigidez, capaz de permitir um fluxo médio de 350 a 450 mL/min e com pontas capazes de minimizar a recirculação do sangue (lúmens com pontas simétricas – por exemplo, Palindrome™ –, Covidien®, assimétricas – por exemplo, Mahurkar™, Covidien® –, ou separadas – Splitcath®, Medcomp®), chamado de maneira geral de permcath. Ambos têm um anel de Dacron® posicionado no interior do túnel subcutâneo, idealmente a 2 cm do orifício de entrada do cateter. Esse anel provoca uma reação inflamatória e consequente aderência, proporcionando melhor fixação do dispositivo após cerca de 1 mês do implante.

**Figura 1:**
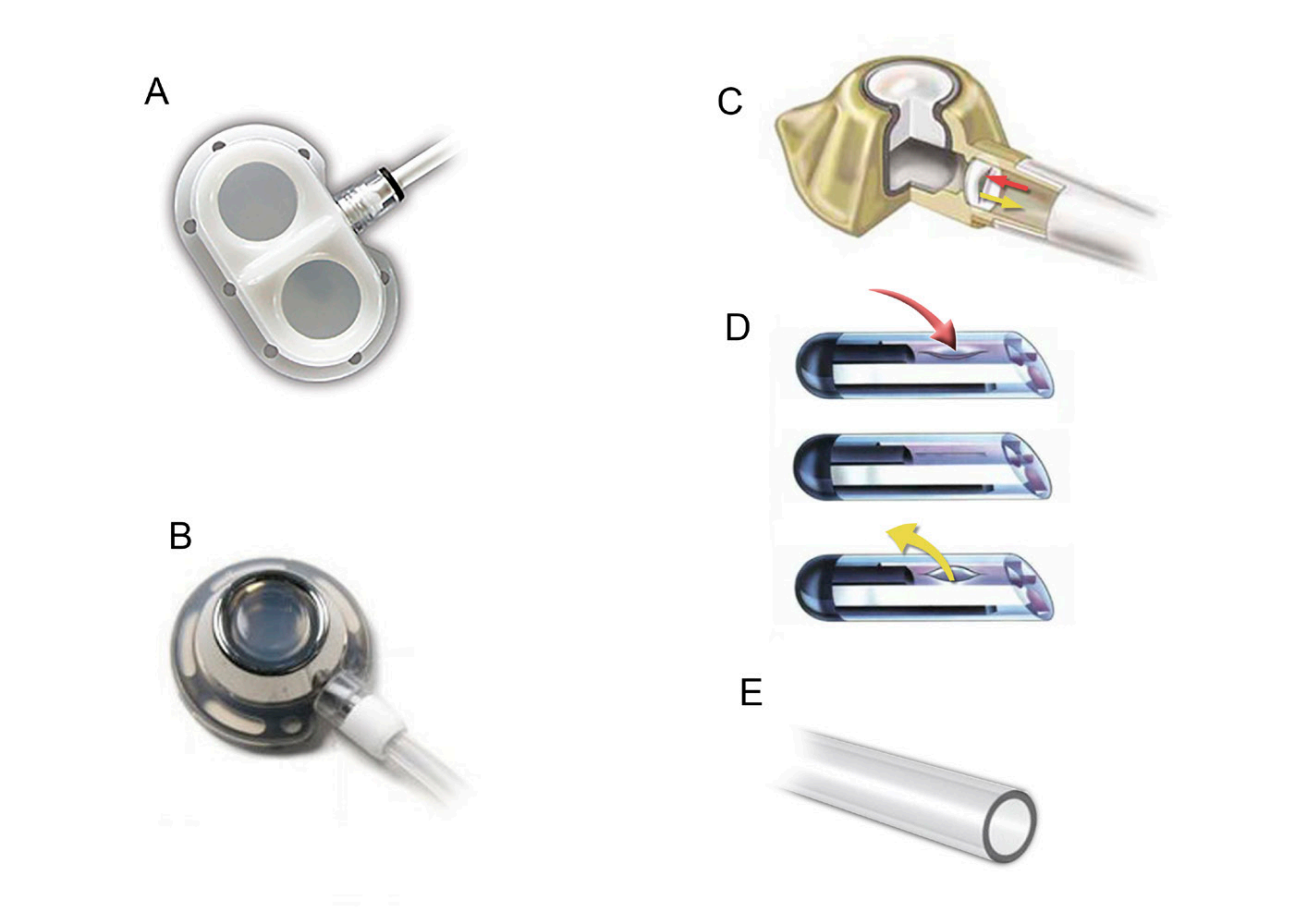
Tipos de cateter totalmente implantável. (A) Câmara dupla de plástico; (B) Câmara simples metálica; (C) Válvula posicionada no reservatório; (D) Válvula na extremidade do cateter de ponta fechada. A pressão negativa abre a válvula, permitindo o refluxo de sangue. Em repouso, a fenda permanece fechada. A pressão positiva, por sua vez, propicia a abertura da válvula e a infusão da medicação; (E) Cateter não valvulado de ponta aberta.

Outro modelo de cateter de longa permanência é o totalmente implantável, conhecido como portocath. Trata-se de cateter com diâmetro inferior a 10 Fr, passível de implantação através de veia periférica ou central e que, após passagem por trajeto subcutâneo, é conectado a um reservatório implantado geralmente sobre a fáscia muscular do local escolhido para a confecção da loja. Como nenhum segmento do conjunto fica exteriorizado, esse tipo de cateter tem menor risco de infecção e maior durabilidade em relação aos semi-implantáveis^18^. O reservatório é fabricado em titânio ou plástico com câmara simples ou dupla (Figura 1). Há dispositivos valvulados e não valvulados. Em alguns modelos, a válvula fica posicionada no reservatório e, em outros, na ponta do cateter (Figura 1). A vantagem dos cateteres valvulados seria diminuir a ocorrência de mau funcionamento causado por trombos intracateter, pelo fato de evitarem refluxo sanguíneo inadvertido. A superioridade dos cateteres valvulados, entretanto, não está comprovada^20^^,^ 21.

Alguns dos novos modelos de cateteres são mais resistentes e permitem a infusão de fluidos com maior pressão (até 5 mL/s, 300 psi), como na injeção por bomba de contraste pelo cateter em exames de imagem (por exemplo, Dignity® - Medcomp, PowerPort® - Bard).

Os cateteres de longa duração (PICC, semi-implantáveis e totalmente implantáveis) são fabricados em silicone ou poliuretano, com diferentes características entre si. Se o silicone mostra melhor biocompatibilidade e menor risco de provocar trombose^22^, o cateter de poliuretano tem paredes mais finas, permitindo maior diâmetro de luz interna em relação a um cateter de mesmo diâmetro externo feito em silicone, o que resulta num menor risco de obstrução^19^.

## INDICAÇÕES DE USO

Os acessos periféricos são os preferidos para infusão de soluções por tempo curto (poucos dias), naqueles indivíduos com rede venosa preservada e para infusão de soluções não vesicantes, que são aquelas que, ao extravasamento, provocam irritação intensa, com formação de lesões bolhosas (vesículas) e necrose tecidual. Pacientes em tratamento quimioterápico não vesicante e por período não prolongado podem beneficiar-se desse tipo de acesso.

O acesso venoso central passa a ser mais indicado que o periférico quando a solução a ser infundida tem pH < 5,0 ou > 9,0, osmolaridade > 500 mOsm/L ou característica vesicante^23^^,^ 24. Necessidade de monitorização da pressão venosa central e impossibilidade de acesso periférico, esta última relativamente frequente em pacientes oncológicos, são outras indicações. O acesso venoso central de curta duração deve ser utilizado apenas em pacientes em regime de internação hospitalar e por tempo inferior a 3 semanas^19^. O PICC pode ser opção nesses casos, principalmente quando o acesso central for necessário por maior tempo (alguns meses) ou o paciente requerer cuidados domiciliares. Atualmente, o PICC tem sido implantado com frequência crescente nos pacientes em quimioterapia ambulatorial, pois permite uso intermitente. Como parte do cateter fica exteriorizada a partir do local de punção, pode causar desconforto.

Os cateteres semi-implantáveis de alto fluxo (permcath) são indicados nos pacientes que necessitam de hemodiálise por período mais prolongado e para indivíduos em programação de aférese, que consiste num processo de coleta de células progenitoras periféricas mobilizadas para a circulação sanguínea após terapia com o fator estimulante de colônias de granulócitos (G-CSF, do inglês *granulocyte colony stimulating factor*), visando transplante de medula óssea. Os cateteres de Hickman permitem a infusão simultânea de diversas soluções, inclusive hemoderivados, além da própria realização do TMO. Além disso, possibilitam a coleta de amostras de sangue para análise, oferecendo conforto por evitar venopunções frequentes, e também a administração de nutrição parenteral prolongada endovenosa^25^.

As principais indicações para a colocação de cateteres totalmente implantáveis são necessidade de acesso venoso frequente, uso de fármacos vesicantes e inadequação do sistema venoso periférico. A utilização desses cateteres requer a punção percutânea do reservatório, motivo pelo qual esses dispositivos são mais indicados para uso intermitente, poupando a pele nos intervalos do tratamento. Sua utilização é quase que exclusiva para o tratamento quimioterápico de pacientes oncológicos^26^.

## TÉCNICAS DE COLOCAÇÃO DOS CATETERES TOTALMENTE IMPLANTÁVEIS

A operação de implante desses cateteres é realizada em ambiente próprio, com o paciente sob monitoração de dados vitais e com suporte de imagem, especialmente de um aparelho de radioscopia. Em geral, essa estrutura é oferecida em centros cirúrgicos e salas de radiointervenção.

O tipo de anestesia depende das condições clínicas do paciente e da preferência da equipe cirúrgica. Geralmente, a anestesia local associada à sedação é suficiente. Por se tratar de operação limpa, não é indicada antibioticoprofilaxia.

A escolha do local de implante deve considerar a veia através da qual será introduzido o cateter e o local em que será criada a loja do reservatório. A preferência é pela introdução em veias que drenam para o sistema cava superior. A inadequação da parede torácica anterior é indicação relativa para implante em veias do sistema cava inferior, uma vez que o reservatório pode ser implantado em locais alternativos, como os membros superiores^27^. A trombose da veia cava superior, entretanto, é indicação absoluta para implante pelas veias safena interna ou femoral^28^. Em situações de exceção, são opções a punção translombar da veia cava inferior, o acesso percutâneo trans-hepático, a canulação de veias colaterais e a recanalização de veias obstruídas^29^^-^ 31.

**Figura 2:**
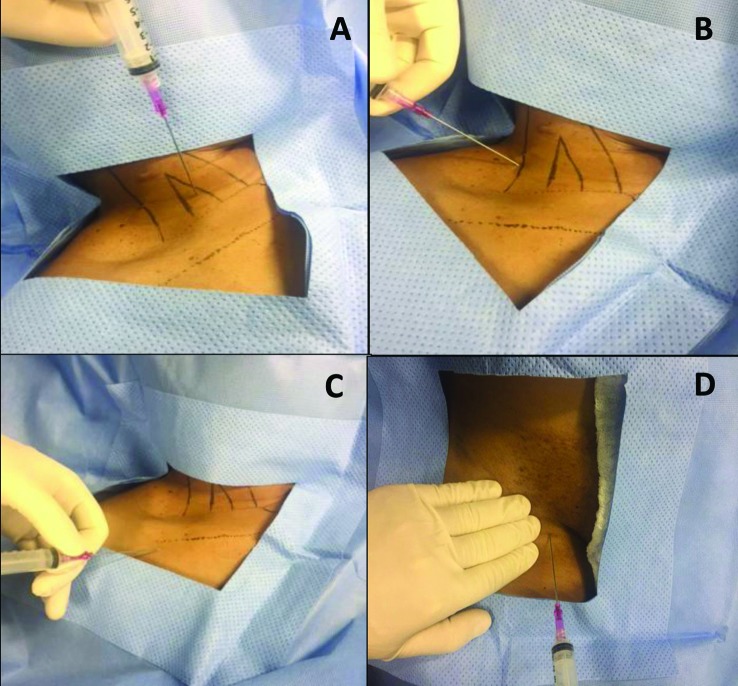
Técnicas de punção das veias profundas mais frequentemente utilizadas para inserção de cateteres venosos. (A) Punção anterior da veia jugular interna (VJI). Entrada entre os ventres do músculo esternocleidomastoideo, com agulha inclinada a 45º em direção ao mamilo ipsilateral; (B) Punção posterior da VJI. Agulha introduzida em direção medial, abaixo do ramo clavicular do músculo esternocleidomastoideo; (C) Punção infraclavicular da veia subclávia com entrada entre os terços médio e lateral da clavícula; (D) Punção da veia femoral realizada medialmente ao local onde é palpado o pulso arterial femoral.

**Figura 3:**
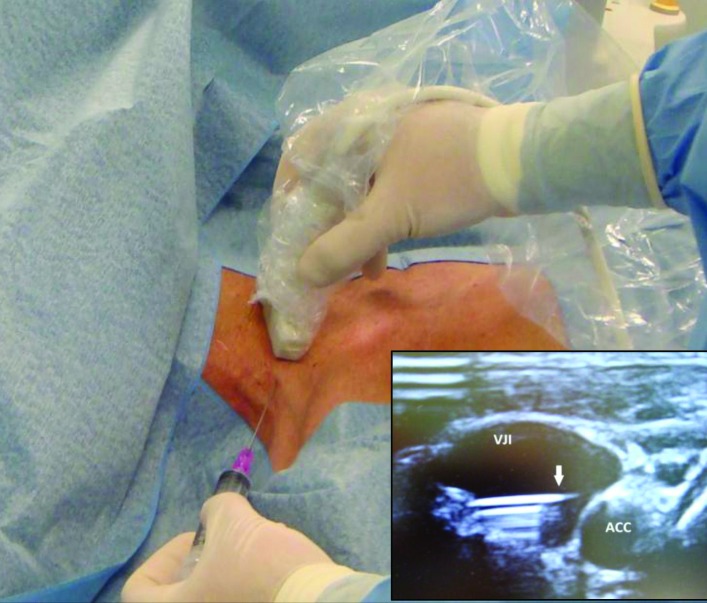
Punção por via posterior da veia jugular interna direita com auxílio do ultrassom. No detalhe, imagem ultrassonográfica da punção mostrando a extremidade da agulha (seta) no interior da veia. VJI: veia jugular interna; ACC: artéria carótida comum.

A técnica de acesso depende do vaso selecionado. Em geral, veias superficiais (jugular externa, cefálica, basílica e safena) são acessadas por dissecção, enquanto as profundas (jugular interna, subclávia e femoral) são abordadas por punção^26^^,^ 32 (Figura 2). O refinamento dos materiais (agulhas, fios-guia) faz com que a punção de veias profundas seja o procedimento de escolha na maioria dos centros. A utilização de ultrassonografia em sala cirúrgica torna possível a avaliação da veia escolhida para punção, permitindo que uma trombose assintomática seja diagnosticada antes do início da operação. Esse recurso permite também a punção guiada por ultrassom, reduzindo riscos de acidentes, como punção arterial e pneumotórax (Figura 3)^33^^,^ 34.

**Figura 4:**
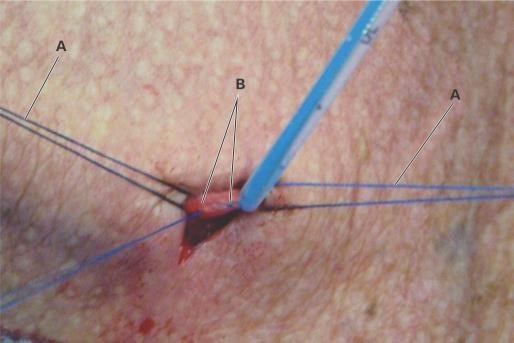
Dissecção de veia jugular externa (VJE) para inserção de cateter de longa permanência. (A) Reparo proximal e distal da VJE; (B) Venorrafia, evitando ligadura distal da VJE e permitindo a manutenção do fluxo pelo vaso.

Quando a opção recai sobre a dissecção de veia superficial, faz-se a venotomia, para que o cateter seja introduzido até que a ponta atinja posição central. O vaso é ligado distalmente, com nova ligadura proximal envolvendo o cateter, tendo-se o cuidado de não provocar a sua constrição. No caso de veias mais calibrosas, uma sutura ao redor da incisão, em substituição à ligadura, permite a manutenção do fluxo sanguíneo, evitando tromboflebites (Figura 4).

O trajeto venoso até o átrio é mais retilíneo à direita, motivo pelo qual a preferência é pela introdução por esse lado. Em caso de tumores na região do tórax (por exemplo, neoplasias de mama), apesar de não haver impedimento para a passagem do cateter pelo mesmo lado, geralmente realiza-se o procedimento no lado contrário ao do tumor.

A extremidade proximal do cateter é deixada na junção átrio-cava, com atenção quanto à ocorrência de arritmias provocadas pelo dispositivo. Em muitos casos, a ponta do cateter pode ficar dentro do átrio direito, sem prejuízo para o paciente.

**Figura 5:**
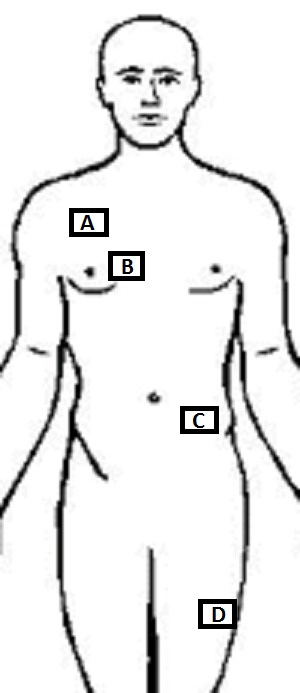
Locais mais frequentes de implante do reservatório. Quando a escolha recai sobre veias que drenam para o sistema cava superior, o reservatório pode ser instalado em posição infraclavicular (A) ou paraesternal (B). Quando da opção pelas veias safena interna ou femoral, o reservatório pode ser posicionado próximo à crista ilíaca anterossuperior (C) ou na face anterolateral da coxa (D).

A loja do reservatório deve ser confeccionada em local firme e distante de áreas em que a pele não seja íntegra, como nas situações em que há estomas, radiodermite ou lesões tumorais ulceradas. Sempre que possível, o reservatório é implantado na parede anterior do tórax, logo acima da fáscia do músculo peitoral (Figura 5). Em pacientes obesos, com tecido celular subcutâneo muito espesso, pode haver dificuldade na punção do reservatório caso este fique alocado sobre a fáscia muscular. Em tal circunstância, a loja do reservatório pode ser feita mais superficialmente, no plano adiposo, deixando-se um mínimo de 2 cm de espessura de tecido subcutâneo sobre o dispositivo.

Quando o acesso se faz através das veias femoral ou safena interna, a loja do reservatório pode ser confeccionada no abdome, medialmente à crista ilíaca anterossuperior, ou na face anterolateral da coxa (Figura 5).

Após o preparo adequado da loja, que compreende hemostasia rigorosa para redução dos riscos de infecção, o cateter é passado por trajeto subcutâneo a partir do local de introdução venosa até a loja. Após essa manobra, realiza-se novo controle radiológico para certificação de que a extremidade do dispositivo se mantém em posição adequada, além de outro teste de fluxo/refluxo. O reservatório é então conectado ao cateter e posicionado no interior da loja, onde é fixado com dois pontos de fio inabsorvível à fáscia muscular. Antes do fechamento do tecido subcutâneo e da pele, faz-se novo teste de fluxo/refluxo, dessa vez puncionando o reservatório, lavando o cateter com o mínimo de 20 mL de solução fisiológica e infundindo solução de heparina antes da retirada da agulha.

## COMPLICAÇÕES ASSOCIADAS AOS CATETERES TOTALMENTE IMPLANTÁVEIS RELACIONADAS AO PROCEDIMENTO DE IMPLANTE

As intercorrências decorrentes do ato operatório de implante são as relativas a acidentes de punção para acesso a uma veia central, como pneumotórax, hemotórax e punção arterial inadvertida, ou à navegação dos dispositivos endovasculares (fio-guia, introdutor, cateter), entre os quais perfuração venosa e lesão miocárdica^35^^,^ 36.

Hematomas e infecções de loja ou trajeto precoces também são eventos adversos que podem estar associados à operação para colocação dos cateteres totalmente implantáveis^26^.

Quando o procedimento é realizado com base em parâmetros anatômicos, o risco de pneumotórax nas punções subclávia e jugular pode chegar a 3%^37^^,^ 38, enquanto punções acidentais de artérias ocorrem em 5% a 10% dos casos^38^. Em alguns estudos, o auxílio da US eliminou a ocorrência de hemotórax e pneumotórax, além de ter-se associado à punção arterial inadvertida com frequência inferior a 1%^39^. Nesses estudos, todavia, ao contrário do nosso, não houve comparação com punções realizadas através de parâmetros anatômicos.

Ocorreram, no serviço dos autores, em 1.255 procedimentos de implante de cateter totalmente implantável, 18 (1,4%) intercorrências relacionadas ao procedimento, entre as quais um (0,1%) pneumotórax e 14 (1,1%) punções arteriais inadvertidas, nove delas em procedimentos guiados por US (0,9% do total)^40^. Nossos dados mostraram que a não utilização da US para guiar a punção venosa é fator de risco para punção arterial iatrogênica mas não para outras complicações, como hemo e pneumotórax^40^.

## COMPLICAÇÕES ASSOCIADAS AOS CATETERES TOTALMENTE IMPLANTÁVEIS RELACIONADAS AO USO DO IMPLANTE

### Complicações infecciosas

As complicações infecciosas são as mais frequentemente relacionadas com cateteres de longa permanência e a principal causa de retirada precoce (antes do final do tratamento) do cateter^26^. A infecção pode ser de loja ou de corrente sanguínea.

#### Infecção de loja

O diagnóstico é feito pelo exame clínico quando há sinais flogísticos (dor, hiperemia, aumento do calor local) na região do reservatório. Pode haver coleção na loja, às vezes acompanhada de deiscência com drenagem de secreção purulenta. O tratamento conservador não costuma trazer bons resultados, levando à retirada do cateter na maioria dos casos, associada à antibioticoterapia sistêmica.

#### Infecção de corrente sanguínea

O diagnóstico de infecção de corrente sanguínea (ICS) ainda é um grande desafio em pacientes com cateteres de longa permanência. Febre e calafrios geralmente estão associados à ICS, mas são sintomas inespecíficos. Quando há suspeita, deve-se obter um par de hemoculturas (HMC) pareadas (aeróbica e anaeróbica) do cateter central e do acesso vascular periférico. O diagnóstico de ICS é estabelecido nas seguintes situações:

- Crescimento do mesmo agente em cultura do cateter e HMC periférica.
- HMC central e periférica positivas:
- *Diferencial de tempo para positividade*: HMC central tem crescimento do microrganismo pelo menos 2 horas antes de HMC periférica.
- *HMC quantitativa*: HMC central tem crescimento do agente infeccioso pelo menos três vezes maior que na HMC periférica.
  - HMC central positiva e HMC periférica negativa.
  - Quando houver sepse sem outro foco infeccioso presumível.

Enquanto se aguarda o resultado das HMC, o tratamento empírico deve incluir cobertura para agentes Gram-positivos e Gram-negativos. Após a identificação do agente infeccioso, deve-se ajustar a terapia conforme o resultado das culturas^41^, mantendo antibiótico sistêmico associado a terapia de selo (lockterapia) por 7 a 14 dias. Após 72 horas de antibioticoterapia eficaz combinada com lockterapia, um novo par de HMC pelo cateter deve ser coletado, independentemente da resposta clínica. Em caso de persistência da positividade para o mesmo agente da infecção, o cateter deve ser removido.

Em pacientes com bacteremia ou fungemia persistentes por até 72 horas após a retirada do cateter, a antibioticoterapia deve ser mantida por 4 a 6 semanas.

Situações que requerem retirada imediata do cateter, sem tentativa de preservação, estão na Tabela 2.

**Tabela 2:** Indicações para retirada do cateter de longa permanência.

Instabilidade hemodinâmica
Hemocultura positiva para *Staphylococcus aureus*, Candida spp
Sepse ou bacteremia persistentes após 48 horas de antibioticoterapia adequada
Complicações sistêmicas (por exemplo., embolia séptica, osteomielite, endocardite)

Além dos cuidados com antissepsia e assepsia no procedimento de implante, há evidências de que a inserção por punção está associada a menor risco de infecção em comparação com a inserção por dissecção venosa^42^. É aconselhável usar dispositivos com câmara dupla somente quando estritamente necessário e manter o cateter para utilização somente em tratamentos quimioterápicos.

As complicações infecciosas na nossa instituição foram as mais frequentes, com prevalência de 13%, 0,35/1.000 dias de uso^40^. A maior parte (66%) dessas intercorrências foi tardia e, portanto, associada ao uso do dispositivo e não ao procedimento de implante^40^.

### Complicações não infecciosas

#### Trombose venosa profunda

Além da possível presença de fatores associados à neoplasia que aumentam os riscos de trombose venosa profunda, como hipercoagulabilidade, lesão endotelial pelo agente quimioterápico e compressão venosa pelo tumor, a presença do cateter pode ser considerada outro fator de risco.

A trombose venosa profunda (TVP) pode gerar sinais e sintomas, tais como dor no trajeto venoso, edema de membro, edema de face e presença de circulação venosa colateral na parede torácica. O diagnóstico é feito através de exames de imagem, como *duplex scan* venoso para os territórios cervical, abdominal e de membros. Se a suspeita for de trombose do tronco braquiocefálico venoso ou de veia cava superior, a angiografia por tomografia computadorizada ou ressonância magnética é mais adequada. Muitas vezes, todavia, o paciente é assintomático e o diagnóstico é feito em exames de rotina realizados durante o tratamento oncológico^43^.

Uma vez estabelecido o diagnóstico de TVP, inicia-se de imediato anticoagulação plena (desde que não haja contraindicação clínica). Pacientes muito sintomáticos, com tromboses extensas, como nos casos de síndrome da veia cava superior, podem ser candidatos a tratamento fibrinolítico, pesando os riscos de complicações hemorrágicas^44^.

Se o cateter mantém o funcionamento adequado, deve ser preservado, uma vez que não há benefício em retirá-lo, além do risco de nova trombose venosa provocada por novo cateter em outro local. A retirada fica restrita aos casos em que o cateter perde o fluxo, o que acontece quando a TVP envolve a extremidade do dispositivo^45^.

Manter a ponta do cateter próxima ou dentro do átrio direito, mesmo nos casos em que o implante é feito por acesso femoral ou safeno, é manobra que contribui para a redução da ocorrência de TVP associada ao cateter.

Entre as complicações não infecciosas registradas na nossa instituição, houve 27 (2,2%) casos de TVP, representando 0,06/1.000 dias de uso^40^. Essa ocorrência é compatível com o que encontramos em outros estudos, nos quais a frequência de TVP variou entre 0,03 a 1,2/1.000 dias de uso^46^. Alguns autores relatam maior risco de TVP no acesso pela veia subclávia em comparação com a introdução via jugular interna^47^^-^ 50, enquanto outros apontam o acesso subclávio como favorável em relação aos outros acessos quanto à incidência de TVP^49^. O acesso femoral também é apontado como de maior risco para TVP em alguns estudos^47^^,^ 48. Nossos dados, entretanto, não mostraram relação entre o sítio de introdução e a ocorrência de TVP^40^.

#### Mau funcionamento

**Figura 6:**
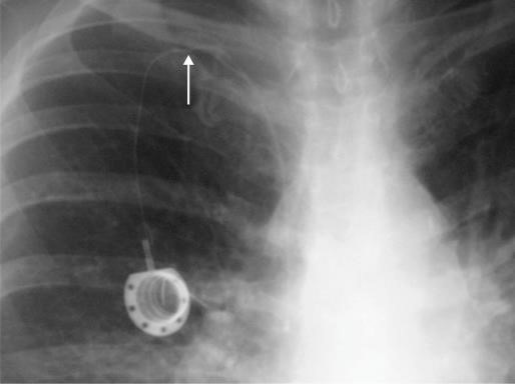
Constrição do cateter (seta) no espaço entre a clavícula e a primeira costela.

Situação na qual ocorre disfunção apenas de refluxo ou deficiência tanto no refluxo quanto na infusão de medicamentos. O mau funcionamento pode ser devido a falha técnica no implante, como posicionamento inadequado da extremidade do cateter, angulação excessiva ou pinçamento do cateter (Figura 6). Essa última situação é mais frequente quando o cateter é passado por punção de veia subclávia, já que o espaço entre a primeira costela e a clavícula é estreito. O mau funcionamento desde as primeiras punções do cateter é indicativo de falha técnica no procedimento de implante^26^.

**Figura 7:**
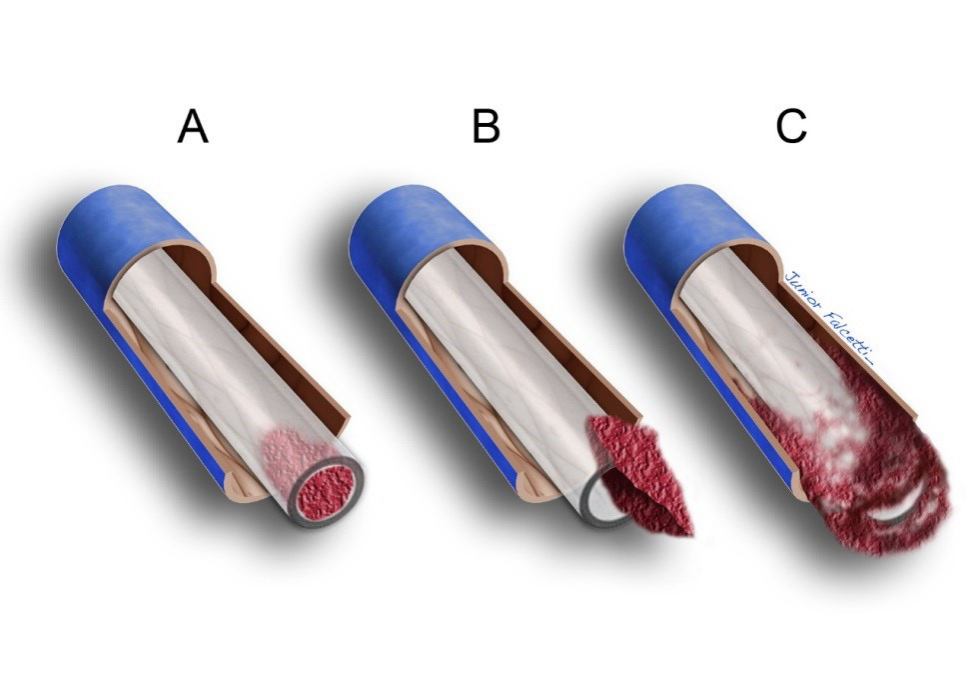
Formação de fibrina na ponta do cateter. (A) Coágulo ou fibrina no interior da luz do cateter; (B) Trombo envolvendo principalmente a área externa do cateter, podendo atuar como mecanismo de válvula, impedindo o refluxo de sangue quando se gera pressão negativa; (C) Trombose envolvendo circunferencialmente a extremidade do dispositivo, obstruindo significativamente a luz do cateter.

A presença do cateter no espaço intravascular pode provocar a formação de fibrina ao seu redor, impedindo o refluxo por atuar como mecanismo de válvula quando se faz pressão negativa durante a aspiração (Figura 7). Nos cateteres com a válvula em fenda na extremidade, a capa de fibrina pode impedir não só o refluxo como também a infusão de fluidos^45^^,^ 51.

Outra condição que traz prejuízo ao funcionamento é a formação de trombos no lúmen do cateter, em decorrência de refluxo de sangue que pode ocorrer, por exemplo, com a pressão negativa quando da retirada da agulha de punção do reservatório^52^.

A avaliação de um cateter com mau funcionamento começa pela checagem da punção. Muitas vezes a deficiência de fluxo se deve à punção inadequada do reservatório. O passo seguinte é a realização de uma radiografia simples de tórax, com a finalidade de avaliar o posicionamento do cateter. A extremidade pode estar mal posicionada devido a falha técnica por ocasião do implante, ou então por migração após um implante adequado. Caso o posicionamento do cateter esteja adequado, sem angulação excessiva e sem sinais de fratura ou pinçamento, pode-se tentar a terapia fibrinolítica, com bons resultados se a disfunção tiver ocorrido há menos de 15 dias^26^.

A TVP pode ser causa de perda da função, se esta envolver a extremidade do cateter^26^.

#### Embolização do cateter

Pode acontecer quando o cateter se desconecta do reservatório, ou então por fratura do cateter, sendo esta última mais frequente nos casos em que o dispositivo é implantado por punção de veia subclávia^45^^,^ 51.

A suspeita é levantada quando o cateter não apresenta refluxo e o paciente queixa-se de dor à infusão de medicamentos. A radiografia simples pode mostrar o cateter desconectado do reservatório ou a fratura completa e possível embolização do cateter. Lesões parciais no cateter não provocam embolização e são diagnosticadas por meio de exame contrastado evidenciando extravasamento do contraste.

Nesses casos, a retirada do dispositivo é mandatória. Se tiver ocorrido fratura completa com migração, pode-se conseguir a remoção do cateter por via endovascular.

#### Rotação do reservatório

A rotação do reservatório faz com que a área de punção fique contra a parede torácica e o fundo virado para cima, impedindo a punção^26^.

A radiografia de tórax em perfil evidencia a rotação de reservatórios metálicos. Se estes forem de material radiotransparente (plástico), a palpação deve ser suficiente para o diagnóstico, já que o reservatório não irá aparecer no exame de imagem.

O tratamento requer reabordagem cirúrgica para reposicionamento e fixação do reservatório.

#### Extrusão do reservatório

A deiscência da pele com exposição do reservatório pode ser decorrente de processo infeccioso, mas também ocorre por necrose da pele, que pode aderir ao reservatório se não houver tecido celular subcutâneo suficiente sobre o dispositivo^53^.

Para evitar essa complicação, deve-se escolher o melhor local para confecção da loja do reservatório, evitando áreas com pouco tecido adiposo, como nas proximidades do manúbrio esternal. Em pacientes caquéticos, deve-se dar preferência ao reservatório de baixo perfil.

#### Falhas do material

Os defeitos primários do dispositivo são raros nos dias atuais, porém ainda podem ser vistos em centros com grande demanda.

Como o tratamento quimioterápico, opção em grande parte dos pacientes oncológicos, é baseado na infusão de drogas endovenosas de maneira intermitente e por período prolongado, cateteres totalmente implantáveis são frequentemente indicados^26^^,^ 28. Tais dispositivos aumentam o conforto e a segurança do tratamento infusional, já que muitas drogas são vesicantes e, não raro, o paciente oncológico apresenta dificuldade no acesso periférico. Desde que manipulados em centros especializados e por equipe de enfermagem treinada no manejo desses dispositivos, os cateteres totalmente implantáveis permitem também a infusão de outros medicamentos por via endovenosa e a coleta de amostras de sangue para análise laboratorial. Essas funções são especialmente úteis quando o paciente está sob regime de internação hospitalar para o tratamento da doença oncológica ou de alguma intercorrência clínica. Modelos mais recentes (por exemplo, Dignity® - Medcomp®, PowerPort® - Bard®) permitem a infusão de soluções através de bombas injetoras, tolerando pressões de até 300 psi e infusões de até 5 mL/seg, o que possibilita a realização de exames como a tomografia computadorizada com injeção de contraste através do cateter.

A manutenção do dispositivo é, portanto, de grande importância para esses pacientes, considerando o longo tempo de tratamento antineoplásico. Dessa forma, a determinação dos fatores de risco para as complicações relacionadas ao cateter de longa duração é essencial para a sua manutenção até o final do tratamento^54^.

Apesar dos avanços em relação à confecção dos cateteres e à técnica operatória^26^^,^ 28, as complicações decorrentes do procedimento de implante e do uso do dispositivo descritas acima continuam a desafiar a equipe multidisciplinar envolvida no tratamento desses pacientes.

Variações quanto à técnica de implante, assim como a ocorrência de complicações e a maneira de lidar com elas, podem estar associadas a questões institucionais, o que deve motivar cada centro oncológico a monitorar a evolução dos seus pacientes portadores de cateteres totalmente implantáveis.
