# Supplementary material for: Cateteres venosos totalmente implantáveis: histórico, técnica de implante e complicações
Source: J Vasc Bras. 2017 Apr-Jun;16(2):128–39. [Article in Portuguese] doi: 10.1590/1677-5449.008216 (PMC5915861; doi:10.1590/1677-5449.008216)
Supplement: Translation

# Translation: Totally implantable venous catheters: history, implantation technique and complications

**Authors:** Antonio Eduardo Zerati, Nelson Wolosker, Nelson de Luccia, Pedro Puech-Leão

**Affiliations:** 1 Universidade de São Paulo – USP, Faculdade de Medicina, Hospital das Clínicas, São Paulo, SP, Brazil.

**Keywords:** historical article, catheters, vascular access devices, infection, ultrasonography, interventional

## Abstract

Access to the venous system is of vital importance for diagnosis and treatment of patients with the most varied range of clinical conditions, whether for taking blood samples or for infusion of solutions. In 1616, Harvey described the circulatory system on the basis of studies in animals and 4 decades later Sir Christopher Wren conducted the first intravenous infusions in living beings. Since then there has been constant evolution in access technique and infusion devices. Of particular note is the creation of long-term catheters in the 1970s, particularly totally implantable devices, which revolutionized cancer treatment, increasing both safety and comfort for oncology patients. The objectives of this article are to review historical data on vascular access and discuss the implantation technique and the main complications associated with procedures for placement and use of totally implantable venous access devices.

## HISTORY

The physiology of blood vessels began to be revealed in the seventeenth century when Harvey, who conducted experiments with animals, published a description of the circulatory system in the 1616 work *Excercitatio Anatomica de Moto Cordis et Sanguinus in Animalibus*.^1^ This knowledge made it possible, a few decades later, to conduct interventions in the blood vessels of living beings, as Folly did in 1654, when he conducted the first blood transfusion between two animals using a silver tube inserted into an artery in the donor and a bone cannula inserted into a vein in the recipient.^2^

In 1656, Sir Christopher Wren, best known as the architect responsible for St Paul’s Cathedral, performed the first infusion into the venous system of living beings, administering, opium, beer, and wine into the veins of dogs, for which he employed a goose quill connected to a pig’s bladder.^3^

In 1663, Robert Boyle and in 1667, Richard Lower described blood transfusions from animals to humans.^4^ The first blood transfusion between human beings did not happen until 1818, when Blundell^5^ transfused to a patient in postpartum hemorrhagic shock blood that had been extracted from a different person.

In 1831, O’Shaughnessy,^6^ and the following year Latta,^7^ successfully treated cholera patients with intravenous infusions of saline solution, and the same principle was described for treatment of people in shock.^8^

The first polyethylene catheter introduced by puncture via the lumen of a needle was created in 1945 and was then released commercially under the name Intracath® (BD Worldwide, Franklin Lakes, New Jersey).^9^

Access to the venous system by puncture was pioneered by the French military surgeon Robert Aubaniac, who described the technique in 1952.^10^ The method he used of puncturing the subclavian vein enabled infusion of greater volumes of fluids more rapidly for treating people in hypovolemic shock on the battlefield. The technique described by Aubaniac involved a medial access, guiding the puncture laterally and inferiorly in the direction of the fossa adjacent to the sternum. Postmortem dissections showed that the point at which the catheters entered the subclavian vein was close to the junction with the internal jugular vein.^10^

In 1952, Seldinger^11^ described intravascular insertion of catheters, advancing them along a flexible guidewire introduced by puncture. This technique remains the basis for procedures used for endovascular access today.

Insertion of central catheters via peripheral veins in the limbs was described in 1960 by Wilson, with the objective of monitoring the central venous pressure of critical patients.^12^

Percutaneous supraclavicular access to the subclavian vein was described in 1965 by Yoffa.^13^ At the time, other techniques for percutaneous catheterization of the internal and external jugular veins were already in use.^3^

Evolution to long-term access routes began in 1973, when Broviac created a silicone catheter that exited via the anterior wall of the thorax after subcutaneous tunneling from the puncture site. The device was synthesized in silicone and included a polyester cuff that provoked an inflammatory reaction, offering better fixation of the catheter by adhesion of the cuff to subcutaneous tissue.^14^

In 1979, Hickman adapted Broviac’s device, creating a new, larger-caliber, model that could be used for plasmapheresis and bone marrow transplantation (BMT).^15^

Another major step in the evolution of vascular accesses was the creation of totally implantable catheters. This technique emerged during the 1970s, after Belin et al.^16^ described implantation of a central venous catheter (CVC) with a subcutaneous chamber for infusion of parenteral nutrition, in 1972. In 1982, Niederhuber et al.^17^ released the results of experiments with 30 totally implantable devices used to treat patients with cancer, 20 of which were placed with the tip in a central venous position, while the remainder were in arterial positions. Such totally implantable catheters are widely used today, primarily for cancer treatment, and are the subject of this article.

## VASCULAR ACCESSES IN CANCER/ONCOLOGY PATIENTS

To ensure that it is capable of offering patients both safety and comfort, the choice of vascular access should take a variety of factors into account, such as definition of which drugs will be administered, the predicted duration of treatment, the frequency with which the access will be employed, the possibility that it will be needed for transfusion of blood products, and the condition of the patient’s peripheral venous network.

## TYPES OF CATHETERS

Different types of venous access can be classified in terms of duration of use, frequency of use, and the site at which the tip is positioned (Table 1).

**Table 1:** Classification of the most widely used types of catheter.

**Catheter**	**Duration**	**Insertion/position of tip**	**Frequency of use**
Jelco®	Up to 4 days	P/P	Continuous
Short-term CVC	Up to 3 weeks	C/C	Continuous
PICC	Up to 12 months	P/C	Continuous/Intermittent
Semi-implantable	Months to years	C/C	Continuous/Intermittent
Totally implantable	Years	C, P/C	Intermittent

P: peripheral; C: central; PICC: peripherally inserted central catheter; CVC: central venous catheter.

Short-duration peripheral catheters are manufactured from teflon or silicone, are around 35 to 52 mm long, and are inserted via puncture of peripheral veins, in a low-risk procedure. They are inexpensive, offer short durability, and are most often used in clinical practice with hospitalized patients.

Short-duration central venous catheters are polyurethane devices of 20 to 30 cm in length and with calibers of up to 8 Fr, that are implanted via puncture of a central vein (internal jugular, subclavian, axillary, or femoral), with the tip positioned close to the cavoatrial junction. There are versions with single or multiple lumens, and they are always for continuous use, exclusively in patients who have been admitted to hospital. Home use is not recommended, because the risks of infection and/or of displacement of the device are greater, because they are not tunneled, and are held fixed in place by a non-absorbable suture to the skin at the entry orifice. The largest-caliber model (12 Fr), known as the Shilley catheter, offers the high flow rates needed for hemodialysis sessions or apheresis, with the drawback that they are short-duration.

Peripherally inserted central catheters (PICCs) are also inserted by puncture of a superficial vein, generally in an upper limb (antecubital, basilic, cephalic), or guided by ultrasonography (US), also by puncture of the brachial vein. These catheters are not tunneled, but they offer long duration and the tip is maintained in a central position. They can be used continuously or intermittently, for treatment at home or in hospital settings. The insertion procedure is low-risk and can be performed at the bedside, foregoing the convenience of controlling advancement of the catheter with imaging. Since these catheters are long (50 to 65 cm in length) and of fine caliber (up to 5 Fr), they are not appropriate for infusion of large volumes in short periods of time. They offer the advantage of ease of removal, but there are disadvantages related to issues of esthetics and comfort.

Tunneled catheters offer greater durability, because the subcutaneous path is a protective factor against infections,^18^ in addition to providing better fixation for the device.^19^ Semi-implantable catheters are introduced via an entry orifice in the skin (generally in the anterior wall of the thorax) and passed via a subcutaneous route to the site of entry into a central vein, from which point they continue in the intravascular space until the tip reaches its position close to the cavoatrial junction. There are two main types of semi-implantable catheters: a more malleable model with symmetrical tips to the lumens (generally two), known as a Hickman line, and a more rigid type that can pass mean flows of 350 to 450 mL/min and has tips designed to minimize recirculation of blood (lumens with symmetrical tips – for example, Palindrome™ –, Covidien®, asymmetrical tips – for example, Mahurkar™, Covidien® –, or separate tips – Splitcath®, Medcomp®), in general known as permcaths. Both types have a Dacron® cuff that is placed within the subcutaneous tunnel, ideally 2 cm from the catheter entry orifice. This cuff provokes an inflammatory reaction, leading to adherence, resulting in better fixation of the device around 1 month after implantation.

Another type of long-stay catheter is the totally implantable version, known as a portacath. These catheters have a diameter less than 10 Fr and can be implanted via a peripheral or central vein and, after taking a subcutaneous path, are connected to a reservoir (port) that is generally implanted over the muscular fascia of the site chosen for construction of the pocket that will accommodate the port. No part of the assembly is outside of the body and so this type of catheter has a lower risk of infection and greater durability than the semi-implantable type.^18^ Ports are fabricated from titanium or plastic and may have single or double chambers (Figure 1). Devices are available with and without valves and in some valved models the valve is positioned in the port and in others it is at the catheter tip (Figure 1). The theoretical advantage of valved catheters is to reduce the occurrence of malfunction caused by intracatheter thrombi, by preventing inadvertent reflux of blood. However, the superiority of valved catheters has not been confirmed.^20^^,^ 21

**Figure 1:** Types of totally implantable catheter (ports). (A) Double-chamber plastic port; (B) Single metal port; (C) Valve located in port; (D) Valve at end of closed-tip catheter. Negative pressure opens the valve, allowing blood to be drawn. At rest, the slit remains closed. Positive pressure, in turn, opens the valve to allow infusion of medications; (E) Open-tip catheter without valve.

Some newer catheter models are more resistant and allow infusion of fluids at higher pressures (up to 5 mL/s, 300 psi), such as injection via the catheter using a contrast pump for imaging exams (for example, Dignity® - Medcomp, PowerPort® - Bard).

Long-term catheters (PICC, semi-implantable and totally implantable) are manufactured from silicone or polyurethane, and each has different characteristics. While silicone offers better biocompatibility and lower risk of provoking thrombosis,^22^ a polyurethane catheter has thinner walls, allowing a larger diameter internal lumen in relation to a silicone catheter with the same external diameter, resulting in a lower risk of obstruction.^19^

## INDICATIONS FOR USE

Peripheral accesses are preferred for short-term infusion of solutions (a few days) in patients with a preserved venous network and for infusion of solutions that are not vesicant. If vesicant solutions leak, they cause intense irritation, formation of boils (vesicles) and tissue necrosis. Patients being treated with non-vesicant chemotherapy for shorter periods can benefit from this type of access.

Central venous access becomes more appropriate than peripheral when the solution to be infused has a pH < 5.0 or > 9.0, osmolarity > 500 mOsm/L, or vesicant characteristics.^23^^,^ 24 Other indications include a need to monitor central venous pressure and factors making peripheral access impossible, which are relatively common in oncology patients. Short-term central venous access should only be used with inpatients and for periods of less than 3 weeks.^19^ In cases for which central access will be needed for longer periods (some months) or the patient must be cared for at home, a PICC is an alternative option. Nowadays, PICCs are increasingly fitted for patients on outpatient chemotherapy, because they allow intermittent use. Since part of the catheter remains outside of the body, exiting via the puncture site, they can cause discomfort.

High flow semi-implantable catheters (permcath) are indicated for patients who require hemodialysis for longer periods and for individuals on apheresis programs, which consists of a process for collecting peripheral stem cells mobilized into the blood circulation after treatment with granulocyte colony stimulating factor (G-CSF), preparatory to bone marrow transplantation. Hickman catheters offer the possibility of simultaneous infusion of different solutions, including blood products, in addition to their use for BMT. They also enable blood samples to be drawn for analysis, thereby offering increased comfort by avoiding frequent vein punctures, and can also be used for administration of prolonged intravenous parenteral nutrition.^25^

The main indications for totally implantable catheters are a need for frequent venous access, use of vesicant drugs, and a peripheral venous system that cannot be used for access. These catheters require percutaneous puncture to access the port, which is why they are more indicated for intermittent use, allowing the skin to recover during intervals in treatment. They are almost exclusively used for chemotherapy treatment of cancer patients.^26^

## TECHNIQUES FOR PLACEMENT OF TOTALLY IMPLANTABLE CATHETERS

The operation to implant one of these catheters is performed in an appropriate setting, in which the patient’s vital signs can be monitored and imaging support is available, particularly fluoroscopy equipment. In general, this infrastructure is found in operating theaters and radiology suites.

The type of anesthesia depends on the patient’s clinical status and the surgical team’s preferences. Generally, local anesthesia combined with sedation is sufficient. Since this is a clean operation, antibiotic prophylaxis is not required.

Choice of the implantation site is based on which vein will be used to insert the catheter and the site in which the port pocket will be created. The preference is for insertion into veins that drain to the superior vena cava system. An anterior chest wall that does not offer adequate conditions is a relative indication for choosing veins of the inferior vena cava system, since the port can be placed in a number of alternative sites, such as the upper limbs.^27^ However, thrombosis of the superior vena cava is an absolute indication for insertion via internal saphenous or femoral veins.^28^ In exceptional situations, alternative options are translumbar puncture of the inferior vena cava, trans-hepatic percutaneous access, cannulation of collateral veins and recanalization of obstructed veins.^29^^-^ 31

The access technique is dependent of the vessel chosen. In general, superficial veins (external jugular, cephalic, basilic, and saphenous) are accessed by dissection, whereas deep veins (internal jugular, subclavian, and femoral) are reached by puncture^26^^,^ 32 (Figure 2). Refinements in materials (needles, guidewires) have resulted in puncture of deep veins becoming the procedure of choice in the majority of centers. Utilization of ultrasonography in the operating room makes it possible to assess the vein chosen for puncture, allowing diagnosis of asymptomatic thrombosis before the operation is started. This resource also enables puncture to be guided by ultrasound, reducing the risk of accidents, such as arterial puncture and pneumothorax (Figure 3).^33^^,^ 34

**Figure 2:** Deep vein puncture techniques most commonly used for insertion of venous catheters. (A) Puncture anterior of the internal jugular vein (IJV). Entry between the bellies of the sternocleidomastoid muscle, with the needle angled at 45º in the direction of the ipsilateral nipple; (B) Puncture posterior to the IJV. Needle inserted in the medial direction, below the clavicular branch of the sternocleidomastoid muscle; (C) Infraclavicular puncture of the subclavian vein, with entry between the medial and lateral thirds of the clavicle; (D) Puncture of the femoral vein medial of the site where the femoral arterial pulse is palpated.

**Figure 3:** Ultrasound guided puncture posterior of the right internal jugular vein. The detail contains the ultrasonographic image of the puncture, showing the tip of the needle (arrow) inside the vein. IJV: internal jugular vein; CCA: common carotid artery.

When the option chosen is dissection of a superficial vein, a venotomy is performed to allow the catheter to be inserted and advanced until the tip reaches the central position. The vessel is ligated distally and a proximal ligature is placed around the catheter, taking care not to constrict it. In the case of larger caliber veins, a suture around the incision, rather than ligature, allows maintenance of blood flow, avoiding thrombophlebitis (Figure 4).

**Figure 4:** Dissection of the external jugular vein (EJV) to insert a long-term catheter. (A) Proximal and distal repair of the EJV; (B) Venorrhaphy, avoiding distal ligature of the EJV, maintaining flow through the vessel.

The venous path to the atrium is straighter on the right, which is why this side is preferred for insertion. In cases in which there are tumors in the thorax area (for example, breast cancer), even if there is no impediment to the catheter’s passage on that side, the procedure is generally conducted contralateral to the tumor.

The proximal extremity of the catheter is placed at the cavoatrial junction, carefully monitoring for possible arrhythmia provoked by the device. In many cases, the tip of the catheter may enter the right atrium, without harming the patient.

The port pocket should be created in site that is firm and is distant from areas in which the skin has lost integrity, such as result from stoma, radiodermatits, or ulcerous tumoral lesions. Whenever possible, the port is implanted in the anterior thorax wall, just above the fascia of the pectoral muscle (Figure 5). In obese patients, very deep subcutaneous tissue could cause difficulties with puncturing the port, if it were placed directly against the muscle fascia. In such cases, the port pocket can be created more superficially, within the adipose plane, leaving subcutaneous tissue a minimum of 2 cm deep over the device.

**Figure 5:** Most frequent sites for placement of the port. When veins that drain to the superior cava system are chosen, the port can be fitted in an infraclavicular (A) or parasternal (B) position. When the internal saphenous or femoral veins are chosen, the port can be placed close to the anterior superior iliac crest (C) or in the anterolateral surface of the thigh (D).

When access is achieved via the internal saphenous or femoral veins, the port pocket can be constructed in the abdomen, medial of the anterior superior iliac crest, or on the anterolateral surface of the thigh (Figure 5).

After the port pocket has been correctly prepared, using rigorous hemostasis to reduce the risks of infection, the catheter is advanced along its subcutaneous path from the vein insertion site to the port pocket. After this, radiology is used once more to ascertain that the tip of the device is still in the correct position and another flow/backflow test is performed. The port is then connected to the catheter and positioned in the pocket, where it is fixed with two non-absorbable sutures to the muscle fascia. Before closure of the subcutaneous tissue and the skin, and another flow/backflow test is performed, this time by puncture of the port, rinsing the catheter with at least 20 mL of saline and infusing a heparin solution before removing the needle.

## COMPLICATIONS ASSOCIATED WITH TOTALLY IMPLANTABLE CATHETERS RELATED TO THE IMPLANTATION PROCEDURE

Intercurrent conditions caused by the operation to implant the device are related to accidents that occur during puncture to access a central vein, such as pneumothorax, hemothorax, and inadvertent arterial puncture, or to navigation of endovascular devices (guidewire, introducer, catheter), which include venous drilling and myocardial injury.^35^^,^ 36

Hematoma and early infections at the pocket or along the catheter path are also adverse events that can be associated with the operation to place totally implantable catheters.^26^

When this procedure is conducted using anatomic parameters as a guide, the risk of pneumothorax caused during subclavian and jugular punctures can be as high as 3%,^37^^,^ 38 while accidental punctures of arteries occur in 5 to 10% of cases.^38^ In some studies, guiding the procedure with US eliminated occurrence of hemothorax and pneumothorax and was associated with an inadvertent arterial puncture rate of less than 1%.^39^ However, in contrast with the present study, these studies did not compare rates with punctures conducted using anatomic parameters.

At our service, in a total of 1,255 procedures to implant totally implantable catheters, there were 18 (1.4%) complications related to the procedure, including one (0.1%) case of pneumothorax and 14 (1.1%) inadvertent arterial punctures, nine of which occurred in US-guided procedures (0.9% of the total number of procedures).^40^ Our data show that not using US to guide venous puncture is a risk factor for iatrogenic arterial puncture, but not for other complications, such as hemothorax and pneumothorax.^40^

## COMPLICATIONS ASSOCIATED WITH TOTALLY IMPLANTABLE CATHETERS RELATED TO USE OF THE IMPLANT

### Infectious complications

Infectious complications are most frequently related with long-term catheters and are the principal cause for early removal (before the end of the treatment) of the catheter.^26^ Infection may occur in the pocket or in the bloodstream.

#### Infection of the port pocket

Diagnosis is by clinical examination when there are phlogistic signs (pain, hyperemia, increased local temperature) in the area of the port. There may be pus build up in the pocket, sometimes accompanied by dehiscence with drainage of purulent secretions. Conservative treatment does not generally achieve good results, and in the majority of cases the catheter has to be removed and systemic antibiotic therapy given.

#### Bloodstream infections

Diagnosis of bloodstream infections (BSI) in patients with long-term catheters is still a serious challenge. Fever and shivering are generally associated with BSI, but these are nonspecific symptoms. When a BSI is suspected, paired blood cultures (BC) should be conducted (aerobic and anaerobic) of samples from the central catheter and from the peripheral vascular access. A diagnosis of BSI is confirmed in the following situations:

- The same infectious agent grows in both the catheter and the peripheral BCs.
- Central and peripheral BCs are positive:
- *Time difference before positive result*: central BC grows a microorganism at least 2 hours before the peripheral BC.
- *Quantitative BC*: central BC has at least three times greater growth of the infectious agent than the peripheral BC.
- Central BC positive and peripheral BC negative.
- There is sepsis with no other presumable focus of infection.

While waiting for the BC results, empirical treatment should cover both Gram-positive and Gram-negative agents. After identification of the infectious agent, treatment should be adjusted to match the culture results,^41^ maintaining systemic antibiotics, combined with lock therapy for 7 to 14 days. After 72 hours of effective antibiotic treatment combined with lock therapy, a repeat pair of BCs should be conducted on samples collected via the catheter, irrespective of the clinical response observed. If there are still positive results for the same infectious agent, then the catheter should be removed.

Patients with bacteremia or fungemia that persists for 72 hours after removal of the catheter should be given antibiotic therapy for 4 to 6 weeks.

Table 2 lists situations that demand immediate removal of the catheter, with no attempt to save it.

**Table 2:** Indications for removal of long-term catheters.

Hemodynamic instability
Blood culture positive for *Staphylococcus aureus*, Candida spp
Sepsis or bacteremia that remain after 48 hours of appropriate antibiotic therapy
Systemic complications (for example, septic embolia, osteomyelitis, endocarditis)

In addition to taking precautions with antisepsis and asepsis during the implantation procedure, there is evidence that insertion by puncture is associated with a lower risk of infection than insertion by venous dissection.^42^ It is also advisable to use double-chamber portals only when strictly necessary and reserve the catheter exclusively for chemotherapy treatments.

At our institution, infectious complications were the most frequent type, with a prevalence of 13%, 0.35/1,000 days of use.^40^ The greater part (66%) of these intercurrent conditions were late and therefore were associated with using the device and not with the implantation procedure.^40^

### Non-infectious complications

#### Deep venous thrombosis

In addition to presence of factors associated with cancer that increase the risks of deep venous thrombosis, such as hypercoagulability, endothelial injury from the chemotherapy agents, and venous compression by the tumor, the presence of a catheter can itself be considered a risk factor.

Deep venous thrombosis (DVT) can cause signs and symptoms such as pain along the path of the vein, edema of a limb, facial edema, and presence of collateral venous circulation in the chest wall. Diagnosis is achieved using imaging exams, such as venous duplex scan of the cervical and abdominal regions and of limbs. If thrombosis is suspected in the venous brachiocephalic trunk or the superior vena cava, then computed tomography angiography or magnetic resonance angiography are more appropriate. However, patients are very often asymptomatic and diagnosis an incidental result of routine tests conducted during cancer treatment.^43^

Once a diagnosis of DVT has been made, full anticoagulation is initiated (as long as there are no clinical contraindications). Highly symptomatic patients, with extensive thromboses, such as cases of superior vena cava syndrome, may be candidates for fibrinolytic treatment, weighing up the risks of hemorrhagic complications.^44^

If the catheter is still functioning correctly, it should be left in place, since there is no benefit from removing it and there is a risk of provoking additional venous thromboses by placing another catheter at a different site. Removal is restricted to cases in which the catheter is no longer patent, which happens when the DVT involves the tip of the device.^45^

The likelihood of occurrence of catheter-related DVT is reduced by maintaining the tip of the catheter close to or within the right atrium, even in cases in which the device is implanted via a femoral or saphenous access.

Among the non-infectious complications recorded at our institution, there were 27 (2.2%) cases of DVT, equating to 0.06/1,000 days of use.^40^ This rate is compatible with what is reported in other studies, in which the frequency of DVT varies from 0.03 to 1.2/1,000 days of use.^46^ Some authors report that the risk of DVT is greater when a subclavian vein access is used, when compared with insertion via the internal jugular,^47^^-^ 50 whereas others state that the subclavian access is better than other accesses in terms of DVT incidence.^49^ Femoral access has also been identified as involving higher risk of DVT in some studies.^47^^,^ 48 However, our data do not show a relationship between site of insertion and occurrence of DVT.^40^

#### Malfunctions

Malfunctions may involve dysfunctions preventing blood drawing only or of both blood drawing and infusion of medications. Malfunction may be the result of technical failure during implantation, such as incorrect positioning of the tip of the catheter, excessive angulation, or pinching of the catheter (Figure 6). The last of these three is most common when the catheter is inserted via puncture of the subclavian vein, since the space between the first rib and the clavicle is narrow. Malfunction immediately after the catheter is first punctured is indicative of technical failure of the implantation procedure.^26^

**Figure 6:** Constriction of the catheter (arrow) in the space between the clavicle and the first rib.

The catheter’s presence in the intravascular space can provoke fibrin formation around it, impeding reflux by acting as a valve mechanism when negative pressure is created during aspiration (Figure 7). With catheters that have a slit-shaped valve at the tip, a fibrin layer may not only prevent blood from being drawn, but also infusion of fluids.^45^^,^ 51

**Figure 7:** Formation of fibrin at the catheter tip; (A) Clot or fibrin inside the catheter lumen; (B) Thrombus primarily involving the external part of the catheter, which may act as a valve mechanism, preventing reflux of blood when negative pressure is generated; (C) Thrombosis enveloping the circumference of the tip of the device, significantly obstructing the catheter lumen.

Another condition that can impact on functioning is formation of thrombi in the catheter lumen, caused by reflux of blood that may occur, for example, when negative pressure is created by removal of the puncture needle from the port.^52^

Investigation of a malfunctioning catheter begins by checking the puncture. Very often failure to achieve reflux is the result of incorrect puncture of the port. The next step is a simple chest X-ray to analyze the position of the catheter. The tip could be misplaced because of technical failure during implantation, or as the result of migration after a successful implantation. If the catheter is correctly positioned, without excessive angulation and with no signs of fracture or pinching, fibrinolysis can be attempted and often produces good results for dysfunctions occurring less than 15 days previously.^26^

A DVT may cause loss of function if it involves the tip of the catheter.^26^

#### Embolization of the catheter

This can occur if the catheter becomes detached from the port or is fractured, which is more frequent in cases in which the device is implanted by puncture of the subclavian vein.^45^^,^ 51

Suspicion is aroused if the catheter will not allow blood to be drawn and the patient complains of pain on infusion of medications. A simple X-ray may show the catheter detached from the port or completely fractured and possible embolization of the catheter. Partially damaged catheters do not provoke embolization and are diagnosed by an examination with contrast in which contrast leakage will be seen.

In these cases, removal of the device is mandatory. If a total fracture with migration has taken place, it may be possible to remove the catheter with endovascular techniques.

#### Port rotation

If the port becomes rotated, the puncture area will be against the chest wall and the base will be facing out, preventing puncture.^26^

A profile chest X-ray can show rotation of metallic portals. However, if the port is made from a radiotransparent material (plastic), palpation should be sufficient for diagnosis, since the port will not be visible on the imaging exam.

Treatment requires a surgical operation to reposition and fix the port.

#### Extrusion of the port

Dehiscence of the skin with exposure of the port can be a result of an infection, but may also be caused by necrosis of skin, which can adhere to the port if there is insufficient subcutaneous tissue over the device.^53^

To avoid this complication, the best available site should be chosen for construction of the port pocket, avoiding areas with too little adipose tissue, such as close to the manubrium of the sternum. In patients with cachexia, low-profile portals should be preferred.

#### Materials failures

Nowadays, primary failures of devices are rare, but can still be observed at high-volume centers.

Chemotherapy is an option for a large proportion of cancer patients and since it is based on the infusion of intravenous drugs intermittently and for prolonged periods, totally implantable catheters are often chosen.^26^^,^ 28 These devices increase comfort and safety of infusion treatments, since many of the drugs used are vesicant and it is not uncommon for cancer patients to have problems that make peripheral access difficult. As long as they are accessed at specialized centers and by nursing teams who have been trained to use these devices, totally implantable catheters also enable intravenous infusion of other medications and drawing of blood samples for laboratory analysis. These functions are especially useful when the patient has been admitted to hospital for treatment of cancer or an intercurrent clinical condition. More recent models (for example, Dignity® - Medcomp®, PowerPort® - Bard®) allow infusion of solutions using injection pumps, accepting pressures up to 300 psi and infusion rates up to 5 mL/s, making it possible to perform examinations such as computed tomography with contrast injected via the catheter.

Maintenance of the device is of great importance for these patients, considering the long duration of antineoplastic treatment. Therefore, determination of the risk factors for complications related to long-term catheters is essential to maintaining them until treatment is complete.^54^

Despite the advances that have been achieved with relation to construction of catheters and operating techniques,^26^^,^ 28 the complications related to implantation procedures and use of the device that have been described above remain a challenge to the multidisciplinary team responsible for treating these patients.

Variations in implantation technique and differences related to occurrence of complications and their management may be related to institutional issues, which should motivate every oncology center to monitor the progress of their patients who have totally implantable catheters.
